# Safety and Effectiveness of Coronary Angiography or Intervention through the Distal Radial Access: A Meta-Analysis

**DOI:** 10.1155/2021/4371744

**Published:** 2021-11-12

**Authors:** Jun Cao, Huaxiu Cai, Weibin Liu, Hengqing Zhu, Gang Cao

**Affiliations:** ^1^Department of Cardiology, Ganzhou Municipal Hospital, Ganzhou 341000, China; ^2^Department of Cardiology, Ganzhou People's Hospital, Ganzhou 341000, China

## Abstract

**Objectives:**

Searching the literature for coronary angiography (CAG) or intervention through distal radial access (DRA) and performing a meta-analysis.

**Background:**

Coronary angiography (CAG) or intervention through distal radial access (DRA) may have a similar success rate, low radial artery occlusion rate, low radial artery spasm rate, and low rate of puncture site hematoma for patients with coronary heart disease. Therefore, the randomized controlled trials (RCTs) were searched, and the data were pooled for meta-analysis to evaluate the effectiveness and safety of DRA.

**Methods:**

RCTs comparing the CAG or intervention through DRA vs. transradial access (TRA) published between January 1, 2017, and May 4, 2021, were searched in the PubMed, Embase, and Cochrane databases. The endpoints included the rate of access success and the number of radial artery occlusions, radial artery spasms, and puncture site hematomas. The data were extracted, and a random-effects model was used for analysis.

**Results:**

Among 204 studies, 6 RCTs (with 2825 participants) met the inclusion criteria. Compared to TRA, the access success rate in DRA (*p*=0.1) and the lower rate of puncture site hematoma were not significantly different (*p*=0.646), while the radial artery occlusion rate (*p* < 0.001) and radial artery spasm rate (*p*=0.029) were significantly lower.

**Conclusion:**

In summary, DRA has a similar access success rate and incidence of hematoma at the puncture site, but a lower incidence of RAO and spasm compared to TRA. These findings demonstrated that DRA is a safe and effective access for CAG or intervention.

## 1. Introduction

Coronary atherosclerotic heart disease (referred to as coronary heart disease in this study) is one of the major cardiovascular diseases threatening human health worldwide; the underlying pathophysiological mechanism is myocardial ischemia and necrosis induced by atherosclerotic stenosis or occlusion of the coronary artery. Myocardial revascularization refers to the removal of coronary artery stenosis and reconstructs blood vessels by coronary artery intervention or surgeries to restore myocardial perfusion. The primary methods of myocardial revascularization include percutaneous coronary intervention (PCI), coronary artery bypass grafting (CABG), and hybrid surgery utilizing both PCI and CABG. With the continuous advancement of techniques and devices, PCI has become a critical method for the treatment of coronary heart diseases. Transfemoral (TF) is the earliest route that can be used for coronary angiography (CAG) and PCI [1]; however, various postoperative complications, including arteriovenous fistula, pseudoaneurysm, severe hemorrhage, and hematoma, have been frequently reported after the processes in recent years. In addition, after the processes through the TF route, immobilization and bedrest are required for the patients. These drawbacks limited the wide application of the TF route in clinical practices. Since the first application of transradial access (TRA) by Kiemeneij et al. in 1993 for PCI [2], this access has been adopted by several interventional cardiologists. The European Society of Cardiology (ESC) has recommended TRA as the preferred access for CAG and PCI in 2013 [3]. Nonetheless, the processes through TRA involve several complications, including radial artery occlusion (RAO) and osteofascial compartment syndrome of the forearm. According to the international consensus published in 2019, several recent studies have shown that despite the preventive measurements (such as increasing the dose of heparin and reducing the time of compression hemostasis) in processes through TRA, the incidence of RAO is still about 3.7% [4]. In clinical practice, repeated CAG or PCI are required for a large number of coronary heart disease patients. In addition, CABG through radial artery is required for some patients with severe coronary heart diseases, and radial artery is used for arteriovenous shunting in some coronary heart disease patients accompanied by uremia, which limits the application of TRA in such patients.

The superficial palmar branch originates from the radial artery at the styloid process of radius, which anastomoses with the terminal ulnar artery to form the superficial palmar arch. After the branching of the superficial palmar branch, the radial artery extends to be the dorsal branch, which enters the anatomical snuffbox and travels through the 1^st^ and 2^nd^ intermetacarpal spaces to the deep palm and anastomoses with the deep palmar branch of the ulnar artery to form the deep palmar arch [5, 6]. The radial artery is termed the distal radial artery after the branching of the superficial palmar branch. The distal radial artery is superficial and with multiple surrounding bony structures, thereby reducing the postoperative compression time [7] and hemorrhagic complications [8]. Due to the presence of a superficial palmar arch, the reduced blood flow rate in the distal artery or occlusion of the distal radial artery does not influence the forward blood flow in the radial artery [9]. Based on the anatomical characteristics of the distal radial artery, several studies suggested that hemostasis might be easier when using the distal radial access (DRA) than TRA, which could reduce the damage to the radial artery and result in a low rate of radial artery occlusion. The diameter of the distal artery is smaller than that of the radial artery, making the puncture rather challenging; therefore, the success rate of the processes through DRA could be lower than through TRA.

In 2017, Kiemeneij et al. [10] first published the observational study of CAG or PCI through DRA and found that the incidence of RAO was low in patients receiving CAG or PCI through DRA; however, several patients needed to convert to other accesses due to puncture failure at the distal radial artery. Similar conclusions have been reported by the subsequent observational studies [11]. In recent several years, several randomized controlled trials (RCTs) demonstrated that compared to TRA, CAG or PCI through DRA have similar access success rate, while the incidence of radial artery occlusion, radial artery spasm, and hematoma at puncture site was lower. Interestingly, the findings of several relevant RCTs are not consistent. Therefore, the present meta-analysis of the available RCTs aimed to obtain the cumulative sample size and consequently increase the statistical power of the data.

## 2. Materials and Methods

### 2.1. Search Strategy

This meta-analysis was performed according to the PRISMA statement. As all the studies on CAG or PCI through DRA were published after 2017 and no such RCTs were published until 2017, studies published in PubMed, Embase, and Cochrane between January 1, 2017, and May 4, 2021, were searched to identify the relevant studies. Both mesh terms and free terms were used to search for PCI. As there were no mesh terms for DRA, the keywords including snuffbox^*∗*^, distal transradial^*∗*^, distal radial^*∗*^, and dorsal radial^*∗*^ were searched in the titles, abstracts, and keywords. The search strategy in the PubMed was as follows: (((“Percutaneous Coronary Intervention” (Mesh)) OR ((Coronary Intervention, Percutaneous^*∗*^) OR (Coronary Interventions, Percutaneous^*∗*^) OR (Intervention, Percutaneous Coronary^*∗*^) OR (Interventions, Percutaneous Coronary^*∗*^) OR (Percutaneous Coronary Interventions^*∗*^) OR (Percutaneous Coronary Revascularization^*∗*^) OR (Coronary Revascularization, Percutaneous^*∗*^) OR (Coronary Revascularizations, Percutaneous^*∗*^) OR (Percutaneous Coronary Revascularizations^*∗*^) OR (Revascularization, Percutaneous Coronary^*∗*^) OR (Revascularizations, Percutaneous Coronary^*∗*^))) AND ((snuffbox^*∗*^(Title/Abstract)) OR (distal transradial^*∗*^(Title/Abstract)) OR (distal radial^*∗*^(Title/Abstract)) OR (Dorsal Radial^*∗*^(Title/Abstract)))) AND ((“2017/01/01” (Date-Publication): “2021/05/04” (Date-Publication))). The search strategy in the Embase was as follows: (“percutaneous coronary intervention”/exp OR “coronary intervention, percutaneous^*∗*^” OR “coronary interventions, percutaneous^*∗*^” OR “intervention, percutaneous coronary^*∗*^” OR “interventions, percutaneous coronary^*∗*^” OR “percutaneous coronary interventions^*∗*^” OR “percutaneous coronary revascularization^*∗*^” OR “coronary revascularization, percutaneous^*∗*^” OR “coronary revascularizations, percutaneous^*∗*^” OR “percutaneous coronary revascularizations^*∗*^” OR “revascularization, percutaneous coronary^*∗*^” OR “revascularizations, percutaneous coronary^*∗*^”) AND (“snuffbox^*∗*^”: ti, ab, kw OR “distal transradial^*∗*^”: ti, ab, kw OR “distal radial^*∗*^”: ti, ab, kw OR “dorsal radial^*∗*^”: ti, ab, kw) AND (1-1-2017)/sd NOT (5-5-2021)/sd. The search strategy in the Cochrane database was as follows: #1 = MeSH descriptor: (Percutaneous Coronary Intervention) explodes all trees; #2 = (Coronary Intervention, Percutaneous^*∗*^) OR (Coronary Interventions, Percutaneous^*∗*^) OR (Intervention, Percutaneous Coronary^*∗*^) OR (Interventions, Percutaneous Coronary^*∗*^) OR (Percutaneous Coronary Interventions^*∗*^) OR (Percutaneous Coronary Revascularization^*∗*^) OR (Coronary Revascularization, Percutaneous^*∗*^) OR (Coronary Revascularizations, Percutaneous^*∗*^) OR (Percutaneous Coronary Revascularizations^*∗*^) OR (Revascularization, Percutaneous Coronary^*∗*^) OR (Revascularizations, Percutaneous Coronary^*∗*^); #3 = #1 or #2; #4 = (snuffbox^*∗*^):ti,ab, kw OR (distal transradial^*∗*^): ti, ab, kw OR (distal radial^*∗*^): ti, ab, kw OR (Dorsal Radial^*∗*^): ti, ab, kw (Word variations have been searched); and #5 = #3 and #4. The references were also scanned manually to identify any eligible studies or relevant reviews.

### 2.2. Inclusion of Studies

The inclusion criteria for the studies were as follows: (1) subjects were patients who received CAG or intervention; (2) the interventional processes were performed through DRA; (3) the control group received processes through TRA; (4) the endpoints were access success rate, RAO, spasm, or hematoma; (5) the study design was RCT. Pseudorandomized studies were excluded from this meta-analysis.

### 2.3. Data Extraction

Two independent investigators (Gang Cao and Hua-Xiu Cai) reviewed the titles and abstracts of the studies retrieved from the database search and evaluated the full-texts of the studies that met the inclusion criteria. The following data were extracted from the included studies and analyzed: country, year of publication, number of subjects, age of subjects, sex of subjects, percentage of 5F or 6F sheath, percentage of smokers, and percentage of patients with diabetes. The disagreements were resolved by discussion with a third investigator (Wei-Bin Liu). The risk of bias was evaluated independently by the two investigators according to the PRISMA statement.

### 2.4. Statistical Analysis

STATA 12.0 software was used for the meta-analysis of data on radial artery occlusion, spasm, and hematoma. The random-effects model (M-H heterogeneity test) was used to estimate the relative risk (RR) of the access success rate, radial artery occlusion, spasm, and hematoma in the study group compared to the control group. Begg and Egger tests were used to evaluate the publication bias (*p* < 0.1 indicated statistical significance). The trim-and-fill method was applied to evaluate the effects of bias on the results. The Cochrane *Q* and *I*^2^ tests were used to evaluate the heterogeneities among the studies, with *I*^2^ > 50% indicating moderate to high heterogeneity.

## 3. Results

**Figure 1:**
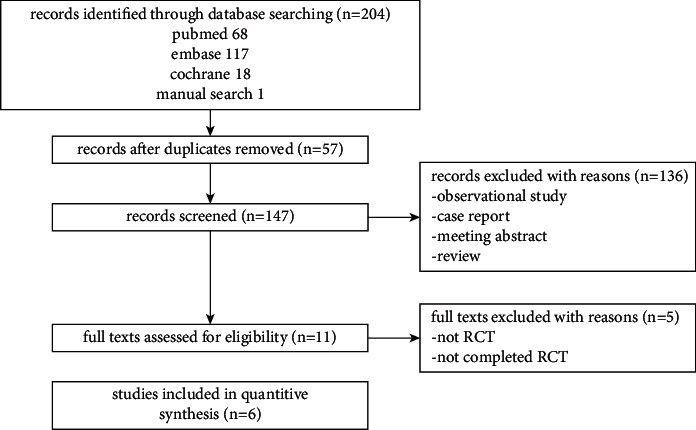
Study selection process.

Finally, 204 studies were retrieved by the search strategy, including 68 from PubMed, 117 from Embase, 18 from the Cochrane database, and 1 from manual search. The studies were imported by the NoteExpress software. Subsequently, 57 duplicates were excluded by NoteExpress. The titles and abstracts of the remaining 147 studies were reviewed, and 136 studies were further excluded. Then, the full-texts of the remaining 11 studies were reviewed, and 5 additional studies were excluded. Finally, 6 studies [8, 12–16] were included in this meta-analysis. The processes of screening are shown in Figure 1.

All the 6 studies were published between 2020 and 2021, and the characteristics of the included studies are given in Table 1. The sizes of sheathes used for the puncture were mainly 6F or 5F, and the average proportion of smokers and patients with diabetes was 22.6% each. The age, sex, size of the sheath, percentage of smokers, and patients with diabetes were similar between the DRA and TRA groups in all the included studies.

The quality of the included studies was evaluated according to the 11 items of Cochrane Back Review Group criteria (Table 2). Two studies reported the randomization sufficiently and 4 studies reported randomization but did not describe the detailed method of randomization. Moreover, 4 studies did not report allocation concealment. The patients in the study and control groups underwent processes through DRA and TRA, respectively, and blinding could not be applied for either the patients or surgeons. During the evaluation of endpoints (including hematoma, radial artery occlusion, and radial artery spasm), the evaluators could also distinguish whether the patients received processes through DRA or TRA, and thus, the evaluators could also not be blinded. Therefore, the score for blinding was 1 point for all the 6 studies. Of these, 1 study described the numbers and reasons of withdrawal/drop off and performed the intention-to-treat (ITT) analysis, while 5 studies did not describe the numbers and reasons of withdrawal/drop off and did not perform the ITT analysis.

**Figure 2:**
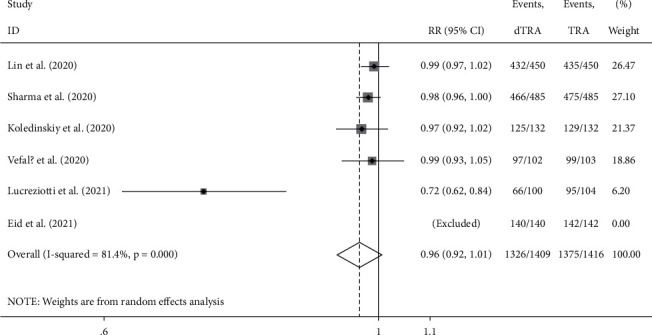
Forest plot (access success).

**Figure 3:**
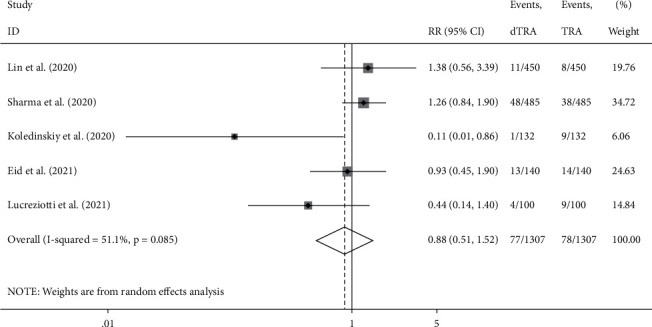
Forest plot (hematoma).

**Figure 4:**
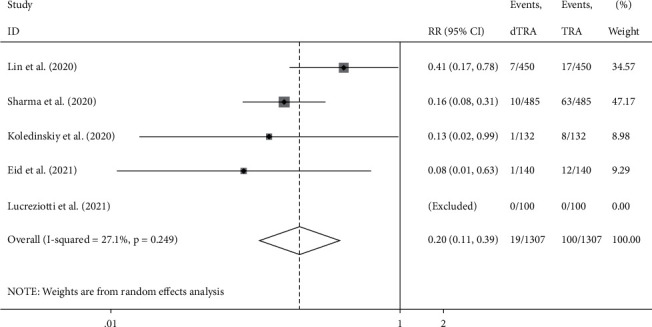
Forest plot (occlusion).

**Figure 5:**
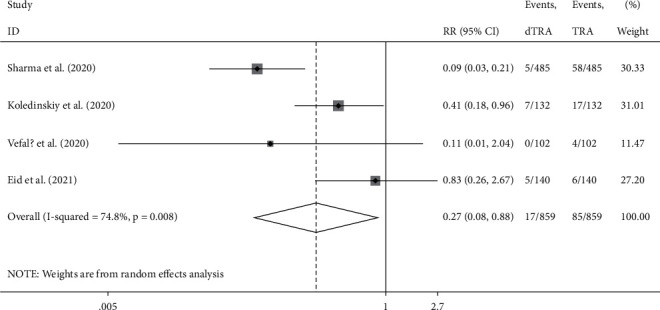
Forest plot (spasm).

Compared to the processes through TRA, the access success rate in DRA was not significantly different (RR: 0.965, 95% confidence interval (CI): 0.924–1.007, *p*=0.1, *I*^2^ = 81.4%); the heterogeneity among the studies was high, and the forest plot is shown in Figure 2. The incidence of hematoma was not significantly different between the two accesses (RR: 0.880, 95% CI: 0.511–1.518, *p*=0.646 , *I*^2^ = 51.1%); the heterogeneity among studies was high, and the forest plot is shown in Figure 3. The incidence of RAO was significantly lower in DRA than TRA (RR: 0.203, 95% CI: 0.106–0.391, *p* < 0.001, *I*^2^ = 27.1%); the heterogeneity among studies was low, and the forest plot is shown in Figure 4. The incidence of radial artery spasm was significantly lower in DRA than TRA (RR: 0.267, 95% CI: 0.082–0.876, *p*=0.029, *I*^2^ = 74.8%); the heterogeneity among studies was high, and the forest plot is shown in Figure 5. As the number of studies included in this meta-analysis was <10, the publication bias was not estimated.

## 4. Discussion

According to the findings reported by Vefalı and Sarıçam [8] and Eid et al. [15], the diameter of the radial artery was 2.32 ± 0.48–2.7 ± 0.4 mm, and the diameter of the distal radial artery was 2.05 ± 0.34–2.4 ± 0.5 mm. Although the diameter of the distal radial artery is smaller than the radial artery and the puncture could be challenging, the distal radial artery is more superficial and has evident anatomical and bony landmarks. In 2019, Sgueglia et al. [17] performed a nonrandomized controlled study in 176 ACS patients (88 in the DRA group and 88 in the TRA group) and found that the access success rate was similar between the two groups (97% vs. 99%). In an observational study performed by Kim et al. [18], data of 138 patients with ST-segment elevation myocardial infarction (STEMI) that received direct PCI were analyzed, and the findings demonstrated that the access success rate was 92.8%, and puncture time was 2.7 ± 1.6 min for DRA. Another observational study in STEMI patients who received direct PCI [19] showed that the access success rate of DRA was 100%, and the average puncture time was 37.36 s. These findings demonstrated that the access success rate of DRA was high, and the puncture time was not increased. The findings of this meta-analysis of RCTs also demonstrated that the access success rate was not significantly different between DRA and TRA.

The superficial palmar branch originated from the radial artery at the styloid process of the radius, which anastomosed with the distal ulnar artery to form the superficial palmar arch. The puncture site in DRA was distant from the radial artery, and thus, the damage to the radial artery is small, and the risks of inducing RAO and spasm were low. The observational and retrospective studies by Kiemeneij et al. [19], Kim et al. [18], and Soydan et al. [19] showed that the incidence of RAO was 0% in DRA. A large-scale retrospective study by Babunashvili et al. [20] showed that the incidence of RAO was 0.61% in DRA. Another observational study by Mizuguchi et al. [21] showed that RAO incidence was 0.4% in DRA. These findings demonstrated that the incidence of RAO was significantly lower in DRA than TRA. The findings of the meta-analysis of these RCTs also demonstrated that compared to TRA, the incidence of RAO was significantly lower in DRA.

Hamandi et al. [22] searched the literature published before April 2019 in the United States National Library of Medicine (NLM), PubMed, and Cochrane Library and compared the incidence of hematoma, as well as radial artery spasm, dissection, and occlusion. Finally, 5 studies (including 4 observational studies and 1 randomized controlled trial) consisting of 6746 patients were included in this meta-analysis. The findings showed that the incidence of hematoma (1.20% vs. 1.24%, RR = 1.01; 95% CI: 0.49–2.07; *p*=0.99), radial artery spasm (1.42% vs. 3.84%, RR = 0.91; 95% CI: 0.32–2.62; *p*=0.86), and radial artery dissection (0.11% vs. 0.20%, RR = 0.63; 95% CI: 0.18–2.16; *p*=0.46) were not significantly different between TRA and DRA, while the incidence of RAO was significantly lower in DRA than TRA (2.30% vs. 4.86%, RR = 0.51; 95% CI: 0.32–0.81, *p*=0.004). Rigatelli et al. [23] searched the literature published before December 22, 2020, in Medline, Scopus, and Web of Science and included 8 case-control studies with 7073 patients in the meta-analysis. The findings demonstrated that compared to CAG or intervention through TRA, processes through DRA had significantly lower RAO incidence (RR: 0.46, 95% CI: 0.31–0.69, *p*=0.002, *I*^2^ = 0%), while the incidence of hematoma (RR: 0.65, 95% CI: 0.37–1.13, *p*=0.12, *I*^2^ = 0%) and radial artery spasm (RR: 0.88, 95% CI: 0.48–1.6 3, *p*=0.001, I^2^ = 0%) was not significantly different. In a systemic review on CAG or intervention through DRA, Coomes et al. [24] searched the literature published before September 2018 in Ovid Medline and Embase and included 4212 patients from 19 studies. The findings showed that the incidence of complications in DRA was 2.4, and the major complication was hemorrhage/hematoma (18.2%), while the incidence of RAO was low in DRA (1.7%). According to the Korea–Europe expert consensus issued in 2021 [25], DRA has been widely acknowledged by experts worldwide in recent several years as new access for CAG and intervention; the anatomical and physiological characteristics could substantially reduce the risk of radial artery occlusion. However, the current evidence is yet limited, and additional large-scale, multicenter RCTs are required to verify the findings.

The meta-analyses by Hamandi et al. [22] and Rigatelli et al. [23] included non-RCTs. Despite the low grade of evidence, the findings still demonstrated that the incidence of RAO was significantly lower in DRA than TRA. The current meta-analysis is the first study of RCTs on DRA, which is high grade of evidence, and the findings were reliable. Therefore, these findings supported the application of DRA in clinical practice and also helped in developing the treatment guidelines on CAG or intervention through DRA.

Nevertheless, the present study has several limitations in this meta-analysis. First, the size of the sheath was associated with radial artery occlusion. Although the sizes of sheaths used in the studies included in this meta-analysis were similar between the DRA and TRA groups, some studies used 6F sheath, while others used 5F sheath, which could influence the results to some extent. Since only 6 studies were included in this study, it was impossible to perform the subgroup analysis. Thus, RCTs are needed for further meta-analysis. Second, several methodological limitations were included in this meta-analysis, which could lead to biases. For instance, 4 studies reported randomization but did not describe the methods of randomization, 4 studies did not report allocation concealment, and 5 studies did not report the numbers and reasons of withdrawal/drop off in detail. Finally, according to the international consensus published in 2019 [4], the incidence of RAO was associated with the time point of evaluation. Among the studies included in this meta-analysis, 5 evaluated RAO before discharge, while 1 evaluated RAO at 30 days after discharge. Therefore, additional RCTs are needed to further investigate the incidence of RAO in the processes through DRA at 30 days after discharge.

## 5. Conclusion

In summary, DRA has a similar access success rate and incidence of hematoma at the puncture site, but a lower incidence of RAO and spasm compared to TRA. These findings demonstrated that DRA is safe and effective access for CAG or intervention.

## Figures and Tables

**Table 1:** General characteristics of the reviewed studies included in the final analysis.

Author	Country	Year	Pts	Age (years)		Male (%)		Sheath (%)		Smoke (%)		DM (%)	
				D	C	D	C	D	C	D	C	D	C
Lin et al.	China	2020	900	55.28	58.81	45.56	50	100 (6F)	100 (6F)	27.56	22.44	10.67	12.44
Sharma et al.	India	2020	970	55	55	60	59	100 (5F)	100 (5F)	NR	NR	NR	NR
Koledinskiy et al.	Russia	2020	264	NR	NR	NR	NR	NR	NR	NR	NR	NR	NR
Vefalı et al.	Turkey	2020	205	60.89	59.84	70.6	68	100 (5F)	100 (5F)	27.5	25.2	36.2	37.8
Eid et al.	Mexico	2021	282	63.1	61.1	75	76.7	88.5 (6F)	92.9 (6F)	20.4	16.9	51.4	43.7
Lucreziotti et al.	Italy	2021	204	NR	NR	NR	NR	NR	NR	NR	NR	NR	NR

Pts, patient's number; C, conventional transradial access; D, distal radial access; DM, diabetes mellitus; NR, not reported.

**Table 2:** Internal validity of the included RCTs^*∗*^.

Study	A	B	C	D	E	F	G	H	I	J	K	Total
Lin et al.	1	0.5	1	1	1	1	1	1	0	1	0	8.5
Sharma et al.	0.5	0	1	1	1	1	1	1	0	1	0	7.5
Koledinskiy et al.	0.5	0	1	1	1	1	1	1	0	1	0	7.5
Vefalı et al.	0.5	0	1	1	1	1	1	1	0	1	0	7.5
Eid et al.	1	1	1	1	1	1	1	1	1	1	1	11
Lucreziotti et al.	0.5	0	1	1	1	1	1	1	0	1	0	7.5

^*∗*^RCT, randomized controlled trial. The internal validity of the included RCTs was assessed by 11 Cochrane Back Review Group criteria: A, the method of randomization was adequate; B, the treatment allocation was concealed; C, the groups were similar in the most important prognostic indicators at baseline; D, the patients were blinded to the intervention; E, the caregivers were blinded to the intervention; F, the outcome assessors were blinded to the intervention; G, cointerventions were controlled; H, compliance was acceptable in all groups; I, the dropout rate was described and acceptable; J, the timing of assessment in all groups was the same; K, ITT analysis was performed. A score of ≥6 indicates a high-quality study.

## Data Availability

The datasets generated and/or analyzed during the current study are available from the corresponding author upon request.
